# Supplementary material for: A multi-modal MRI study of the central response to inflammation in rheumatoid arthritis
Source: Nat Commun. 2018 Jun 8;9:2243. doi: 10.1038/s41467-018-04648-0 (PMC5993749; doi:10.1038/s41467-018-04648-0)
Supplement: Supplementary file 1 — Supplementary Information [file 41467_2018_4648_MOESM1_ESM.pdf]

Supplementary Information

A multi-modal MRI study of the central response to inflammation in rheumatoid arthritis

Schrepf, Kaplan, et al.

Supplementary Table 1 Clinical Characteristics for 54 rheumatoid arthritis patients

	Baseline visit	6 month visit
	Mean, s.d.	
RA disease activity[a]	3.62±1.30	3.39±1.36
ESR (mm/hr)	19.39±14.66	17.76±14.92
CRP (mg/L)	7.78±8.54	7.52±10.81
Fatigue[b]	4.59±2.19	4.91±2.01
Number of painful sites[d]	6.48±4.64	6.59±3.94
Pain[f]	3.81±2.38	2.91±1.92
Cognitive performance (%)[g]	82.48±18.71	84.79±16.45

[a]Disease activity score 28; [b]Current fatigue 0-10 numerical rating scale (NRS);Widespread Pain Index of the 2011 fibromyalgia survey; Current pain NRS; [g] percentage of correct paced auditory serial addition test responses

Supplementary Table 2 Frequency and percentage of treatment with anti-inflamamtory drugs

Medication<br><br>N (%)	Baseline	6 month visit
Methotrexate	29 (54)	30 (56)
Sulphasalazine	6 (11)	6 (11)
Lefunomide	5 (9)	7 (13)
Hydroxychloroquine	5 (9)	5 (9)
Rituximab	7 (13)	7 (13)
Tociluzimab	4 (7)	5 (9)
Adalumimab	7 (13)	7 (13)
Etanercept	7 (13)	6 (11)
Certoluzimab	7 (13)	8 (15)

Supplementary Table 3 The network based statistic inflammation configuration

Edge	Node 1	Network	Node 2	Network
13 to 28	13	Uncertain	28	Sensory/somatomotor Hand
13 to 34	13	Uncertain	34	Sensory/somatomotor Hand
13 to 47	13	Uncertain	47	Cingulo-opercular Task Control
25 to 116	25	Sensory/somatomotor Hand	116	Default mode
28 to 264	28	Sensory/somatomotor Hand	264	Dorsal attention
34 to 47	34	Sensory/somatomotor Hand	47	Cingulo-opercular Task Control
34 to 264	34	Sensory/somatomotor Hand	264	Dorsal attention
39 to 47	39	Sensory/somatomotor Hand	47	Cingulo-opercular Task Control
39 to 112	39	Sensory/somatomotor Hand	112	Default mode
39 to 202	39	Sensory/somatomotor Hand	202	Fronto-parietal Task Control
39 to 213	39	Sensory/somatomotor Hand	213	Salience
39 to 215	39	Sensory/somatomotor Hand	215	Salience
39 to 216	39	Sensory/somatomotor Hand	216	Salience
43 to 177	43	Sensory/somatomotor Mouth	177	Fronto-parietal Task Control
43 to 212	43	Sensory/somatomotor Mouth	212	Salience
43 to 215	43	Sensory/somatomotor Mouth	215	Salience
47 to 48	47	Cingulo-opercular Task Control	48	Cingulo-opercular Task Control
49 to 116	49	Cingulo-opercular Task Control	116	Default mode
55 to 177	55	Cingulo-opercular Task Control	177	Fronto-parietal Task Control
68 to 82	68	Auditory	82	Default mode
68 to 177	68	Auditory	177	Fronto-parietal Task Control
69 to 177	69	Auditory	177	Fronto-parietal Task Control
70 to 177	70	Auditory	177	Fronto-parietal Task Control
75 to 199	75	Default mode	199	Fronto-parietal Task Control
75 to 256	75	Default mode	256	Dorsal attention
75 to 260	75	Default mode	260	Dorsal attention
86 to 177	86	Default mode	177	Fronto-parietal Task Control
86 to 207	86	Default mode	207	Salience

86 to 227	86	Default mode	227	Subcortical
87 to 208	87	Default mode	208	Salience
87 to 259	87	Default mode	259	Dorsal attention
90 to 190	90	Default mode	190	Fronto-parietal Task Control
95 to 190	95	Default mode	190	Fronto-parietal Task Control
95 to 192	95	Default mode	192	Fronto-parietal Task Control
95 to 258	95	Default mode	258	Dorsal attention
116 to 144	116	Default mode	144	Visual
116 to 165	116	Default mode	165	Visual
116 to 190	116	Default mode	190	Fronto-parietal Task Control
116 to 192	116	Default mode	192	Fronto-parietal Task Control
116 to 197	116	Default mode	197	Fronto-parietal Task Control
116 to 199	116	Default mode	199	Fronto-parietal Task Control
116 to 200	116	Default mode	200	Fronto-parietal Task Control
116 to 256	116	Default mode	256	Dorsal attention
116 to 258	116	Default mode	258	Dorsal attention
116 to 259	116	Default mode	259	Dorsal attention
116 to 264	116	Default mode	264	Dorsal attention
122 to 150	122	Default mode	150	Visual
122 to 227	122	Default mode	227	Subcortical
150 to 206	150	Visual	206	Salience
151 to 187	151	Visual	187	Fronto-parietal Task Control
151 to 192	151	Visual	192	Fronto-parietal Task Control
160 to 187	160	Visual	187	Fronto-parietal Task Control
177 to 215	177	Fronto-parietal Task Control	215	Salience
196 to 206	196	Fronto-parietal Task Control	206	Salience

All identified connections and putative networks from the network based statistic analysis of higher erythrocyte sedimentation rate and whole brain connectivity. Nodes are identified by their parcellation according to the Power Atlas.

Supplementary Table 4 Eigenvector centrality hubs at baseline

ROI	Approximate structure
13	L precuneus
15	L SMA paracentral lobule
25	R postcentral
26	R postcentral
28	R precentral
30	L postcentral
31	R SMA medial frontal
32	R postcentral
34	L postcentral
40	R SMA medial frontal
47	L SMA medial frontal
53	R SMA sup frontal
54	R SMA medial frontal
59	L mid cingulate
89	R precuneus
94	mid cingulate
112	L frontal sup medial
113	L dorsal ACC
134	L precuneus
135	R precuneus
136	R precuneus
157	R mid occip
159	R cuneus
166	L precuneus
174	L precentral
188	L mid frontal BA
190	R IPL
191	L IPL superior
192	R IPL
194	R IPL
195	L IPL
199	R IPL
202	L medial sup frontal
203	R PCC paracentral mid cing
204	R supramarginal

205	R middle frontal precentral
213	L mid cing SMA
214	L mid frontal
215	midline dorsal ACC
216	R mid cing
251	R precuneus
255	R postcentral
256	R precuneus
258	R sup parietal
260	L precuneus mid occip
261	L mid frontal precentral
263	L sup parietal
264	R mid frontal

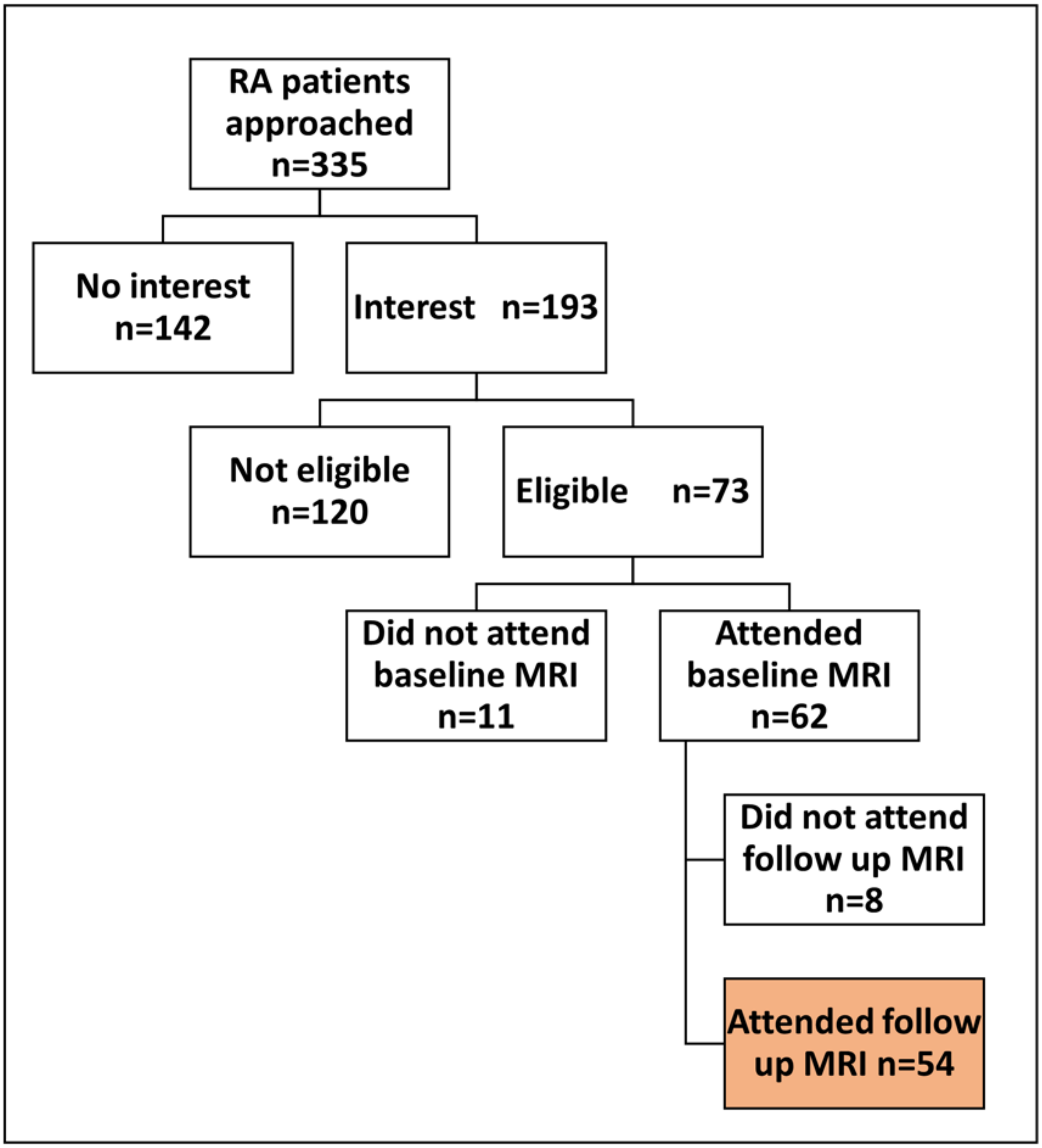

Supplementary Figure 1. Study flow chart.
